# Distal-less induces elemental color patterns in *Junonia* butterfly wings

**DOI:** 10.1186/s40851-016-0040-9

**Published:** 2016-03-01

**Authors:** Bidur Dhungel, Yoshikazu Ohno, Rie Matayoshi, Mayo Iwasaki, Wataru Taira, Kiran Adhikari, Raj Gurung, Joji M. Otaki

**Affiliations:** The BCPH Unit of Molecular Physiology, Department of Chemistry, Biology and Marine Science, University of the Ryukyus, Okinawa, 903-0213 Japan

**Keywords:** Butterfly wing, Color pattern, Distal-less, Eyespot, GFP, *Junonia orithya*

## Abstract

**Background:**

The border ocellus, or eyespot, is a conspicuous color pattern element in butterfly wings. For two decades, it has been hypothesized that transcription factors such as Distal-less (Dll) are responsible for eyespot pattern development in butterfly wings, based on their expression in the prospective eyespots. In particular, it has been suggested that Dll is a determinant for eyespot size. However, functional evidence for this hypothesis has remained incomplete, due to technical difficulties.

**Results:**

Here, we show that ectopically expressed Dll induces ectopic elemental color patterns in the adult wings of the blue pansy butterfly, *Junonia orithya* (Lepidoptera, Nymphalidae). Using baculovirus-mediated gene transfer, we misexpressed Dll protein fused with green fluorescent protein (GFP) in pupal wings, resulting in ectopic color patterns, but not the formation of intact eyespots. Induced changes included clusters of black and orange scales (a basic feature of eyespot patterns), black and gray scales, and inhibition of cover scale development. In contrast, ectopic expression of GFP alone did not induce any color pattern changes using the same baculovirus-mediated gene transfer system.

**Conclusions:**

These results suggest that Dll plays an instructive role in the development of color pattern elements in butterfly wings, although Dll alone may not be sufficient to induce a complete eyespot. This study thus experimentally supports the hypothesis of Dll function in eyespot development.

## Background

Butterfly wing patterns are highly diverse and represent suitable targets for evolutionary developmental biologists [1–3]. Complex butterfly wing color patterns are believed to be constructed by modifications of the nymphalid groundplan [1, 4–7]. The nymphalid groundplan is composed of color pattern elements that belong to distinct symmetry systems. Among the butterfly color pattern elements, eyespots are probably the most conspicuous to predators and humans alike. Actual eyespot patterns in nymphalid butterflies are highly diverse in shape and coloration [8], but an ideal eyespot is concentric with serial rings of different colors, as found in *Bicyclus* and *Junonia* butterflies.

It has been two decades since genes putatively involved in eyespot development, such as *Distal-less* (*Dll*), were identified based on their expression in locations corresponding to future eyespots [9, 10]. Subsequent gene expression pattern studies revealed additional genes and led to speculation that networks of transcription factors play important roles [11–16]. In one study, *Dll* sequence variation was linked to eyespot size variation [17]. However, plasticity of eyespot size may be controlled by other factors, as a subsequent study suggested that eyespot size variation in seasonal morphs may arise from the temporal expression variation of *Notch* and *engrailed* but not *Dll* [18]. Furthermore, distinct eyespots in some nymphalid butterflies lack *Dll* expression [19, 20]. These results suggest that Dll is involved in eyespot development in many, but not all, nymphalid butterflies, and that Dll is not a universal regulator of eyespot determination in butterflies.

Recently, it has been reported that non-specific overexpression of *Dll* throughout the body in a transgenic butterfly, *Bicyclus anynana*, resulted in an increase of eyespot size and an increased number of eyespots [21]. RNAi knockdown induced by heat shock resulted in a decrease in the size of the inner black ring, but not a decrease of the number of eyespots [21]. Thus, it has been proposed that Dll plays a role in eyespot size determination [21], supporting the previous study of Dll function in that process [17]. A recent study that used a novel method to correlate eyespot size with the *Dll* expression level has reached a similar conclusion; *Dll* contributes to eyespot size determination, but weakly and in a non-exclusive fashion [22], indicating that eyespot size is likely regulated by Dll and other factors.

Additionally, in the same transgenic study [21], laser heat-shock-induced up-regulation of *Dll* in a restricted wing region resulted in black patches, but not eyespots [21]. Although negative controls did not show black patches [21], one possible interpretation is that the black patches were induced by a toxic effect of Dll overexpression, as black scales are readily induced by physical damage [23–26] and likely by other types of cellular damage as well [27–29] with up-regulation of endogenous *Dll* [14]. Above all, the failure of the experimental up-regulation of Dll to induce elemental color patterns besides a simple black patch [21] may be interpreted as a negative evidence against the hypothesis that Dll plays an important role in eyespot formation. In contrast, if black scales were truly induced by the functional activity of Dll, that could indicate that Dll is able to induce an entire element, but simply because of low expression levels or because of other unknown reasons, only a fragment of an element (i.e., black scales) was induced. To resolve this issue, we believe that the functionality of *Dll* in eyespot development should be investigated further. The available data on the possible roles of *Dll* in eyespot development must be complemented by other methodologies.

As a complementary strategy, we focused on baculovirus technology [30]. We developed a baculovirus-mediated gene transfer method for butterfly wings, which involves injections of a recombinant baculovirus and anti-gp64 antibody [31]. In that study, we successfully transferred and expressed *green fluorescent protein* (*gfp*) driven by the polyhedrin promoter in the pupal and adult wings of the blue pansy butterfly *Junonia orithya*. Importantly, GFP expression did not affect the adult wing color patterns at all [30]. It has been reported that expression of GFP under the polyhedrin promoter is detectable as early as 12 h post-infection [32]. Because the candidate genes for eyespot formation are expressed from the late larval to early pupal stages [9–16], and because ectopic eyespots can be induced by physical damage to early pupal wings [23–26], we reasoned that Dll overexpression in a wing tissue (more precisely, position-dependent misexpression or ectopic expression within a wing tissue) at the early pupal stage, mediated by a recombinant baculovirus vector, could induce elemental color patterns if Dll is functional.

In the present study, we engineered a recombinant baculovirus vector harboring a fusion gene, *Dll-gfp*, whose expression was driven by the polyhedrin promoter in infected cells. With this strategy, we tested if Dll can induce any element-like structures in butterfly wings and if Dll expression is sufficient for the production of eyespots. We successfully obtained ectopic expression of the Dll-GFP fusion protein in the developing pupal wings that led to ectopic elemental color patterns but not a complete eyespot. On the basis of these results, we discuss possible roles of Dll in color pattern development of butterfly wings.

## Methods

### Experimental design

The objective of this study is to examine the functions of Dll in developing butterfly wings. To do this, a *Dll-gfp* fusion gene was transferred to living wing tissues through a baculovirus vector. Our hypothesis was that if Dll is sufficient for eyespot development, ectopically expressed Dll should be able to construct ectopic eyespots or similar elemental color patterns.

### Butterflies

We used the blue pansy butterfly *J. orithya* (Linnaeus, 1758). Female adult individuals were field-caught in Okinawa-jima or Ishigaki-jima in the Ryukyu Archipelago, Japan. This is a common butterfly in this region, and no permission is required to catch them in the field. Eggs were collected from these females. Alternatively, larvae were caught on these islands. Larvae were fed their natural host plants at ambient temperature (25–27 °C). Pupae were also placed at the same ambient temperature (25–27 °C) before and after experimental treatments.

### Baculovirus design and production

We designed a recombinant *Dll-gfp* baculovirus vector that contained an expression unit for *Dll* (*J. coenia Dll* sequence: GenBank Accession No. AF404110.1) and green fluorescent protein (*Aequorea victoria gfp* sequence: GenBank Accession No. L29345.1). The construct was *Dll*-spacer-*gfp*-His6-stop (1131 + 24 + 714 + 18 + 3 = 1890 bp; GenBank Accession No. KP748528).

The entire baculovirus production process based on this sequence information was performed by Wako Pure Chemical Industries, Ltd. (Osaka, Japan). First, the entire construct was chemically synthesized with the flanking *Xba* I sequence at the 5’ end and *Bgl* II sequence at the 3’ end as a part of a plasmid pBMH. The construct was excised and subcloned into the cloning site of *Xba* I and *Kpn* I of a transfer vector pPSC8 (Protein Sciences, Meriden, CT, USA). Purified transfer vector (2 μg), linear baculovirus (AcNPV) DNA (85 ng), and Insect GeneJuice Transfection Reagent (5 μL) (Merck, Darmstadt, Germany) were mixed with Sf900II SFM (200 μL) (Gibco, Life Technologies, Carlsbad, CA, USA). The mixture was added to a 25 cm^2^ flask with 1.0 × 10^6^ Sf9 cells. The cells were incubated at 28 °C for 6 days. The supernatant was collected as co-transfection medium. This co-transfection medium (1/200 of the culture volume) was added to infect *express* SF^+^ cells (1.5 × 10^6^ cells/mL in Sf900II SFM) in a 100 mL culture in a 250 mL flask. This was incubated for 72 h at 28 °C with shaking (130 rpm). The culture medium was collected and centrifuged (3000 × *g*, 4 °C for 30 min). The supernatant, approximately 1 × 10^7^ pfu/mL as estimated by a conventional plaque assay, was stocked for pupal injections.

The supernatant and pellets were subjected to SDS-PAGE and Western blot analysis using an anti-His antibody conjugated with horseradish peroxidase, Penta · His HRP (QIAGEN, Hilden, Germany). The blot signals were detected using Immobilon Western Chemiluminescent HRP Substrate (Millipore, Billerica, MA, USA). As predicted, the expressed Dll-GFP protein was clearly detected from the pellet and not from the supernatant (not shown). Dll-GFP was not secreted to liquid media from the infected cells.

In addition to the *Dll-gfp* baculovirus, we used a control *gfp* baculovirus that was obtained from AB Vector (San Diego, CA, USA) at the original baculovirus titer of 1 × 10^8^ pfu/mL. In these *gfp* and *Dll-gfp* baculovirus vectors, gene expression was driven by the strong polyhedrin promoter [30, 32], and thus we assumed that GFP or Dll-GFP was expressed immediately after infection as early as 12 h post-infection [32].

### Pupal injections

For each baculovirus vector, the baculovirus dilution factor for injection and the post-infection time for antibody injection were optimized for the present study. This is partly because the *Dll-gfp* baculovirus vector appeared to be more toxic than the *gfp* baculovirus vector. Injection site was always located at the abdominal segments 5 or 6, which are considerably remote from pupal wings. This ensured that no physical damage on pupal wings was elicited during an injection process. We note that only heavy physical damage can induce ectopic patterns; accidental physical damage, if any, on wings during the injection process does not induce ectopic patterns.

For the *Dll-gfp* baculovirus vector, pupae were injected through the abdominal cuticle as mentioned above with 2.0 μL of a solution containing the recombinant baculovirus vector within 18–24 h after pupation using an Ito microsyringe (Fuji, Shizuoka, Japan). At the same site, 6 h post-infection, we injected 2.0 μL of mouse monoclonal anti-gp64 antibody IgG_2a_ (200 μg/mL in PBS) against the baculovirus gp64 (AcV1) of extracellular nonoccluded AcNPV (*Autographa californica* nucleopolyhedrovirus) (Santa Cruz Biotechnology, Santa Cruz, CA, USA) using an Ito microsyringe (Fuji, Shizuoka, Japan). The 18–24 h post-infection injection of antibody caused 100 % pupal mortality with 2, 5, 10, 100, and 1000 fold dilutions of baculovirus (although not with 10,000-fold dilutions). However, using 100- and 500-fold dilutions of the *Dll-gfp* baculovirus (1 × 10^5^ and 2 × 10^4^ pfu/mL; 2.0 μL) and anti-gp64 antibody injection (2.0 μL) 6 h post-infection, we were able to obtain GFP fluorescence from adult wings.

For the *gfp* baculovirus, we followed the original protocol [31] with some modifications; the diluted *gfp* baculovirus (1 × 10^6^ pfu/mL or less; 2.0 μL) was injected 18-24 h post-pupation, followed by an injection of anti-gp64 antibody (2.0 μL) 18–24 h post-infection.

These gene transfer experiments were permitted by the Safety Committee for Genetic Recombination Experiments of the University of the Ryukyus.

### Visualization of GFP fluorescent signals

When necessary, pupal wings from 4-day-old pupae were dissected following a published protocol with some modifications [33]. The pupa was lightly anesthetized on ice. The cuticle around the wing margin was cut using a scalpel and lifted up to cut through the trachea connecting the wings to the thorax. Dissected wing tissues were placed on glass slides and then directly subjected to the fluorescent microscope to examine GFP fluorescence.

Whole pupae, whole adults, isolated pupal wings, or isolated adult wings were placed on the ATTO illuminator VISIRAYS-B (Tokyo, Japan), a blue-LED light unit with emission wavelengths λ = 440–500 nm and λ_max_ = 470 nm. Under this illuminator, low magnification GFP fluorescence images were observed and recorded using a Canon digital single-lens reflex camera EOS 50D (Tokyo, Japan) with an ATTO filter SCF515.

For high magnification images of GFP fluorescence, we used a Nikon inverted epifluorescence microscope Eclipse Ti-U (Tokyo, Japan) equipped with a Nikon Intensilight C-HGFI (a mercury pre-centered fiber illumination system with a 130-W Hg lamp), a Nikon Epi-Fl Filter Cube GFP-B (EX480/40, DM505, and BA535/50), and a Hamamatsu Photonics ImagEM EM-CCD camera (Hamamatsu, Japan). This microscope hardware system was controlled with a Hamamatsu Photonics AQUACOSMOS/RATIO analysis system. For these observations, we either isolated pupal wings or lifted the forewing to expose the surface of the hindwing as described elsewhere [22, 25].

For bright-field low-magnification images, we used a Canon digital single-lens reflex camera EOS 50D (Tokyo, Japan) and a Saitou Kougaku microscope SKM-S30-PC (Yokohama, Japan). For bright-field high-magnification images, we used a Keyence high-resolution digital microscope VHX-1000 (Osaka, Japan) and the Nikon microscope system described above.

### Detection of transcripts

Pupae were injected with the *Dll-gfp* baculovirus followed by anti-gp64 antibody. Three days after anti-gp64 antibody injection, pupal wings were dissected according to a standard protocol [33]. To compare *Dll* gene expression levels between infected and non-infected individuals, we used 4-day-old infected and non-infected pupae. We also used non-infected first-day pupae for comparison. Isolated wings were readily frozen at –80 °C. The RNeasy Kit (QIAGEN) was used for RNA isolation. Total RNA was isolated from both the right and left dissected fore- and hindwings of three treated pupae (with 100-fold or 500-fold diluted baculovirus vectors) or three non-treated pupae. The isolated total RNA (340 ng per reaction) was subjected to RT-PCR using the AccessQuick RT-PCR System (Promega, Madison, USA) with AMV reverse transcriptase (Promega) and *Tfl* DNA polymerase (Promega).

The thermal cycling conditions for detecting the *Dll-gfp* transcript were 45 °C for 45 min, 95 °C for 2 min, 45 cycles of 95 °C for 1 min, 50 °C for 30 s, and 72 °C for 2 min, and lastly 72 °C for 5 min, using DLL2UP primer 5’-AAGTCTGCGTTCATAGAGTTACAGC-3’ and GFPDOWN primer 5’-GTATAGTTCATCCATGCCATGTGTAATC-3’. The expected size of the amplified DNA was 1708 bp.

To detect the *Dll* mRNA transcripts without *gfp* transcribed from the endogenous genomic DNA and from the baculovirus-mediated transgenes, RT-PCR was performed under thermal cycling conditions as follows: 45 °C for 45 min, 95 °C for 2 min, and 20 cycles of 95 °C for 1 min, 50 °C for 30 s, and 72 °C for 1 min 20 s, and the last incubation at 72 °C for 5 min. For *Dll*, DLL1UP primer 5’-ATGACCACCCAGGAGCTAGATCACC-3’ and DLL1DOWN primer 5’-AGGGTTGGCATCAGCCTGGTACCAG-3’ were used for the first round of PCR. Nested PCR was then performed with *Tfl* DNA polymerase using the DLL2UP primer described above and DLL2DOWN primer 5’-TACTGCGGCACGTAGGGCGGGTGCG-3’. Thermal cycling conditions for the nested PCR were set as follows: 95 °C for 2 min, 25 cycles of 95 °C for 1 min, 50 °C for 1 min, and 72 °C for 1 min 20 s, and the last incubation at 72 °C for 5 min. The expected size of the amplified DNA was 855 bp. The PCR products were subjected to electrophoresis using 1 % Agarose S (NIPPON GENE, Tokyo, Japan) with ethidium bromide (Promega).

After subjecting the PCR products to electrophoresis, the band intensities were measured and compared semi-quantitatively. Agarose gel images were taken with Image Quant LAS 400 (GE Healthcare Life Sciences, Piscataway, USA) and were used for image analysis using Image Quant TL 7.0 400 (GE Healthcare Life Sciences). A given gel was imaged three times, the band intensities were measured for each image, and their mean values were used as a final value for that gel. To compare *Dll* gene expression levels between infected and non-infected individuals, we used 4-day-old infected and non-infected pupae. We also used non-infected first-day pupae for comparison.

### Statistical analysis

To examine the difference in expression levels, we performed two-sided Student’s *t*-test using IBM SPSS Statistics 19 (2010). To examine the difference in occurrence of ectopic color patterns between the two baculovirus constructs, Fisher’s exact test was performed using JSTAT 13.0 (2012).

## Results

### GFP expression in pupae

**Fig. 1:**
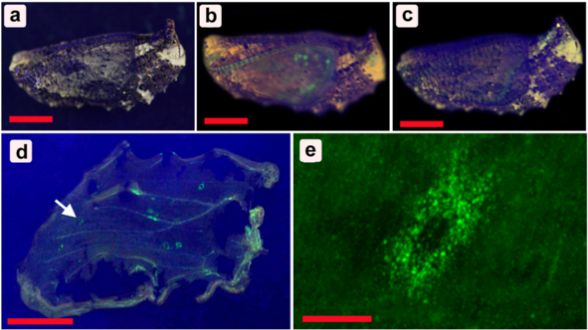
Fluorescent signals from the Dll-GFP fusion protein expressed in pupal wings after baculovirus-mediated gene transfer. **a** A non-treated pupa under the blue illuminator. Scale bar, 5 mm (also applicable to **b**-**d**). **b**, **c** The *Dll-gfp* baculovirus-treated pupae under the blue illuminator. Green areas signify GFP fluorescent signals. **d** An isolated forewing that exhibits many patches of GFP fluorescence. An arrow indicates a single GFP-positive patch that is enlarged in **e**. **e** A GFP-positive patch. Numerous small green dots are epithelial cells expressing Dll-GFP. Scale bar, 200 μm

After the *Dll-gfp* baculovirus vector injection, Dll-GFP protein expression was verified in 11 individuals with GFP fluorescence in their pupal wings from among 289 pupae in our optimized conditions (i.e., 2 μL of 100- or 500-fold diluted baculovirus solution injected 6 h post pupation) using a whole-mount illuminator and confocal microscope (Fig. 1). These GFP-positive signals were detected as early as the fourth day post-pupation.

**Fig. 2:**
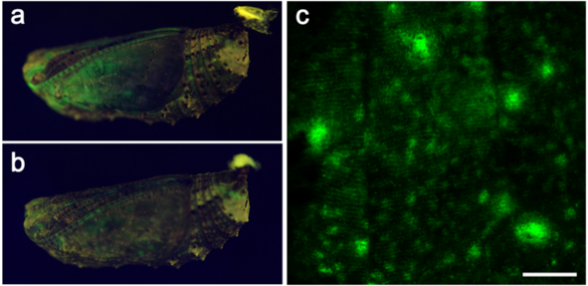
GFP fluorescent signals from pupae treated with the *gfp* baculovirus vector. **a**, **b** Pupae with GFP expression on wings under the blue illuminator. **c** High-magnification live image of a GFP-positive pupal hindwing *in vivo* 24 h post-antibody treatment. The dorsal hindwing was exposed by a surgical procedure and observed with confocal microscopy. Aligned green epithelial cells expressing GFP are observed. Scale bar, 300 μm

As a control, we injected the *gfp* baculovirus vector. We readily obtained GFP signals (in various parts of the body) from 137 individuals of 515 treated pupae (Fig. 2). Thus, 26.6 % (137/515) of the treated individuals were GFP-positive in pupae. The GFP fluorescent levels of these pupae (based on visual inspections) were similar to those of the previous study [31]. Comparatively, the GFP signals with the *Dll-gfp* baculovirus appeared to be lower than those with the *gfp* baculovirus. This may be because Dll protein is located in nucleus, or because the Dll protein is toxic to differentiating cells if highly expressed. Alternatively, it may be simply due to the larger size of Dll-GFP compared with GFP, resulting in a lower production rate and intramolecular inhibition of GFP fluorescence by the Dll protein portion.

**Fig. 3:**
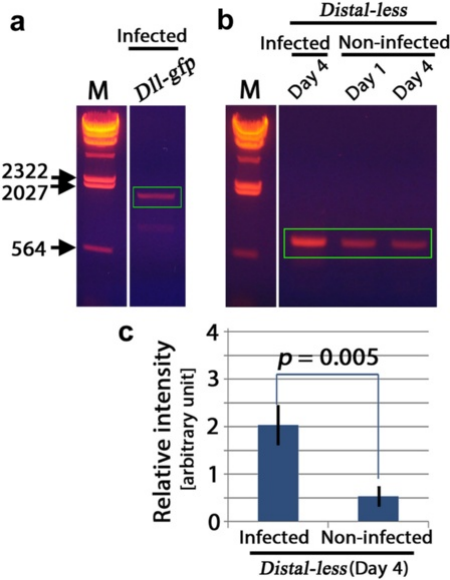
Detection of *Dll-gfp* mRNA from wings by RT-PCR. **a** The *Dll-gfp* cDNA amplified from wings of infected pupae (a boxed band), which corresponds to the predicted cDNA size, 1708 bp. M: λHindIII marker. These two lanes were run together in a single gel. **b** *Dll* cDNA amplified from wings of infected and non-infected pupae (boxed bands) corresponding to the predicted cDNA size, 855 bp. Pupal wings were isolated at the post-infection day indicated. Endogenous *Dll* is amplified in addition to exogenous *Dll-gfp*. M: λHindIII marker. These four lanes were run together in a single gel. **c** Semi-quantification of *Dll* mRNA transcripts (including exogenous and endogenous ones) (*n* = 3 for each category) from 4-day-old pupal wings. The data shown are mean ± SD

To verify that fluorescent signals were detected from GFP molecules, not from autofluorescence and other unknown factors (although we confirmed no autofluorescence from non-infected pupae), and to confirm the expression of the intact fusion mRNA, the wings of the treated pupae were isolated at the fourth day post-pupation, and the RNA samples were subjected to RT-PCR (*n* = 2). The cDNA for *Dll-gfp* (1708 bp) was detected as predicted (Fig. 3a), demonstrating the integrity of the *Dll-gfp* mRNA. These results thus indicate that the green fluorescent signals from the fourth day pupae originates from Dll-GFP.

The level of *Dll* transcript at the fourth day post-pupation was also examined by RT-PCR in which both the *Dll* region of the exogenous *Dll-gfp* transcript from the baculovirus construct and the endogenous *Dll* transcript from the native gene were amplified (*n* = 3) (Fig. 3b). It appeared that *Dll* was expressed endogenously, even at the fourth day post-pupation (Fig. 3b).

To compare the exogenous *Dll-gfp* with the endogenous *Dll* expression, we performed semi-quantitative image analysis of the RT-PCR products. Compared with the non-treated pupae, we found an approximately 3.8-fold increase of *Dll* mRNA in infected pupae (*p* = 0.005, unpaired two-sided Student’s *t*-test) (Fig. 3c).

These results based on GFP florescence and RT-PCR demonstrated successful expression of the *Dll-gfp* construct in the pupal wings.

### Efficiency for color pattern changes

In the adult wings of the individuals infected with the *Dll*-*gfp* baculovirus, ectopic element-like color patterns were found in five individuals, three of which were GFP-positive at the ectopic sites and two of which were not, after the treatment of 289 individuals (number of dead individuals at the pupal stage with no color pattern development = 39; number of dead individuals at the pupal stage with color pattern development but no eclosion = 177; number of individuals that eclosed = 73). It is important to stress that GFP signals from adult wings, which are not composed of live cells, are extraordinary, and it was not surprising to have seen no GFP signals from the ectopic sites on adult wings. Indeed, even in the two individuals that did not show GFP signals at the ectopic sites on adult wings, GFP signals were observed at the pupal stage. Twenty-three individuals showed GFP signals (in various parts of the body) at the pupal stage and developed adult color patterns inside the pupal case. Most individuals died before the completion of color patterns or eclosion, although some of these were GFP-positive. Therefore, 8.0 % (23/289) of the treated individuals were GFP-positive and completed adult color patterns, and 1.7 % (5/289) of the treated individuals were positive for ectopic color patterns, indicating that 21.7 % (5/23) of the GFP-positive individuals showed treatment-induced ectopic color patterns.

**Fig. 4:**
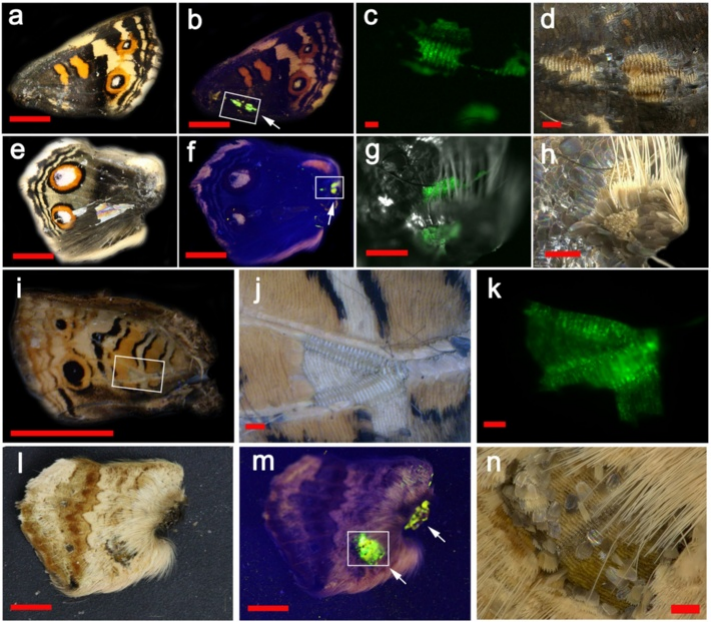
GFP expression in adult wings after the treatment with the *gfp* baculovirus vector. **a**, **b** A dorsal forewing under the bright-field illuminator (**a**) and under the blue illuminator for GFP (**b**). A GFP-positive area is boxed and indicated by an arrow. Scale bars, 5 mm. **c**, **d** High-magnification images of a GFP-positive area boxed in **b**. Scale bars, 200 μm. **e**, **f** A dorsal hindwing under the bright-field illuminator (**e**) and under the blue illuminator (**f**). A GFP-positive area is boxed and indicated by an arrow. Scale bars, 5 mm. **g**, **h** High-magnification images of a GFP-positive area boxed in **f**. Scale bars, 200 μm. **i** A ventral forewing. Boxed area is shown in **j** and **k**. Scale bar, 5 mm. **j**, **k** High-magnification images of **i**. Scale bars, 200 μm. **l**, **m** A ventral hindwing under the bright-field illuminator (**l**) and under the blue illuminator (**m**). GFP-positive areas are indicated by arrows; one area is boxed for higher magnification. Scale bars, 5 mm. **n** High-magnification image of a GFP-positive area boxed in **m**. Scale bar, 200 μm

As described above, we injected the *gfp* baculovirus and obtained 137 individuals with GFP-positive signals (in various parts of the body) as a control (Fig. 2), but did not observe any color pattern modifications or necrotic damage in these GFP-positive individuals in adult wings (Fig. 4). Similar results (no color pattern changes and necrotic damage after GFP expression) have already been reported [31]. However, we noted that many individuals showed GFP fluorescence from the wing basal membrane, where scales were absent. This could mean that scale development was impaired by GFP expression or by the baculovirus infection itself, although we cannot exclude the possibility that physical damage during the wing isolation process caused scale removal at given GFP-positive sites.

Fisher’s exact test revealed that the difference between the *Dll-gfp* baculovirus (i.e., five color patterns changed in 23 GFP-positive individuals) and the *gfp* baculovirus (i.e., no color patterns changed in 137 GFP-positive individuals) was statistically significant (*p* < 0.0001; two-sided Fisher’s exact test). This result is consistent with our previous finding that the *gfp* baculovirus infection causes no color pattern changes [31] and showed a significant contribution of the overexpressed Dll to color pattern changes in the treated wings.

### Case analysis of ectopic patterns

**Fig. 5:**
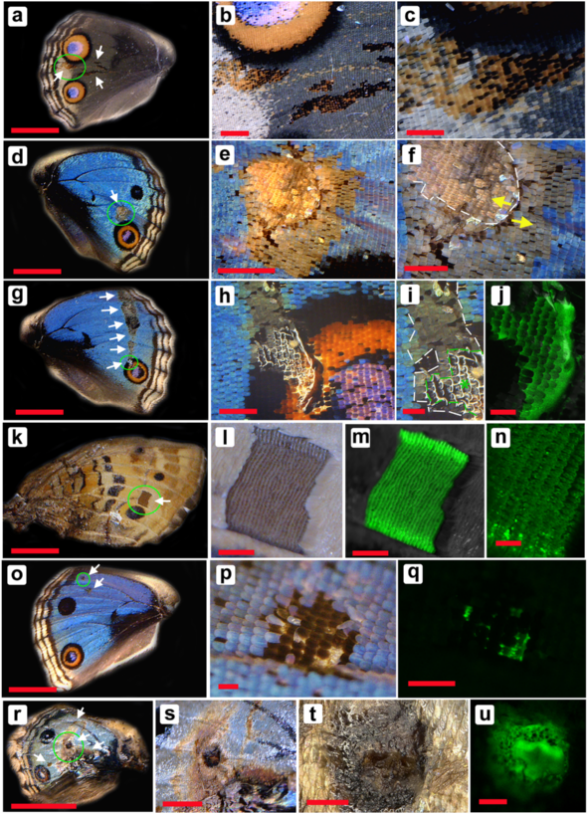
Dll-induced ectopic color patterns. White arrows indicate ectopic patterns. Areas in green circles in the left panels are enlarged in subsequent panels. **a**-**c** Black and orange mosaic clusters of scales. Scale morphology and size are normal, but colors are not. Color quality of these modified scales is similar, if not identical, to that of the nearest eyespot. Scale bars: 5 mm (**a**), 1 mm (**b**), and 500 μm (**c**). **d**-**f** Orange and black (gray) mosaic cluster of scales. Colors are not as vivid as those in **a**-**c**, but the mosaic pattern is similar. Some scales show reversed direction, as indicated by yellow arrows and white broken line in **f**. Scale bars: 5 mm (**d**), 1 mm (**e**), and 500 μm (**f**). **g**-**j** Black and gray clusters of scales. Transparent scales are observed in an area superimposed on the large eyespot. The modified area is surrounded by white broken lines in **i**. The area with transparent scales is indicated by green broken lines and an asterisk in **i**, from which GFP fluorescent signal was obtained, as indicated in **j**. The GFP signal is observed in the basal membrane but not the scales themselves. Scale bars: 5 mm (**g**), 500 μm (**h**), and 200 μm (**i**, **j**). **k**-**n** GFP-positive rectangular cluster of gray scales. As shown in **m** and **n**, GFP signal is observed from the scales themselves. Scale bars: 5 mm (**k**), 500 μm (**l**, **m**), and 200 μm (**n**). **o**-**q** Black spots. The basal membrane but not scales shows GFP fluorescence. Scale bars: 5 mm (**o**) and 200 μm (**p**, **q**). **r**-**u** GFP-positive necrosis-like damage. A GFP-positive black patch is observed. In addition, gray bands that contain undeveloped scales are observed. Scale bars: 5 mm (**r**), 1 mm (**s**), and 200 μm (**t**, **u**)

We analyzed the five cases that exhibited clear ectopic color patterns (Fig. 5). In the first case, we found patchy expression of the black-orange clusters that did not alter the morphology and size of scales (Fig. 5a–c). In this species, normal orange scales are found exclusively in the light (non-dark) ring of an eyespot in this dorsal surface of the wing, and the orange scales are associated with black scales in an eyespot. The ectopic black-and-orange cluster may thus be equivalent to a fragment of an eyespot.

In the second case, a relatively large orange area was found containing black and gray scales that were aligned somewhat like a circular element (Fig. 5d–f). These scale colors were similar to, but less vivid than, those of the first case. This may be because the scales also developed fine structures for blue structural color by default. Surprisingly, in the central area of the ectopic structure, the orientation of scales was reversed (Fig. 5d–f). We did not detect GFP fluorescence from the color-modified scales or their basal membrane in the first and second cases. The failure to detect GFP in the scales was not surprising, as the adult scales were filled with dark pigments that prevent GFP detection and the scales are extracellular constructs that remain after the death of scale cells. However, GFP expression was confirmed at the pupal stage, although a direct positional correspondence between ectopic patterns in adult wings and GFP fluorescence at the pupal stage was impossible to confirm; these ectopic patterns were positioned in the hindwings.

In contrast to the large ectopic orange area found in the second case, the third case with color pattern changes involved large clusters of dark black and gray colors that crossed the hindwing from the apex to the eyespots (Fig. 5g–j). These colors were similar to those detected in the second case, despite the lack of orange color. The black scales were more abundant in the peripheral region of the ectopic elements, reminiscent of eyespots. The ectopic pattern was partially superimposed on the normal eyespot, where some cover scales appeared to be transparent, possibly lacking pigments, and a portion of basal membrane was exposed. GFP fluorescence was detected from this pigment-less area. This may be because the state of transcription factors in developing scale cells is resistant to cellular interpretation. In addition, black scales were induced within the normal orange band.

The fourth case of color pattern changes exhibited a rectangular gray elemental structure with a sharp boundary just above a normal eyespot (Fig. 5k–n). In this case, the ectopic scales themselves (not the basal membrane) were GFP-positive. Detection of GFP fluorescence directly from scales was rare, even with *gfp* baculovirus infection [31]. The detection of GFP fluorescence probably occurred because this particular wing was freshly isolated from the pupal case.

The fifth case developed relatively small black spots (Fig. 5o–q). This induction may be melanization of cover scales, but the impaired development of the blue cover scales that resulted in the exposure of the black ground scales cannot be excluded. GFP fluorescence was detected in the basal membrane around the black spot, confirming that the black spot was not produced by accidental physical removal, but by ectopic Dll-GFP expression.

Consistent with this possibility, we observed another eight cases of abnormal wing patterns; however, these were likely produced by necrosis. In a particular individual, a black patch was GFP-positive (Fig. 5r–u). In addition, a large gray band of undeveloped scales was observed. Therefore, we obtained 13 cases of abnormal patterns in total. However, eight necrotic cases and five non-necrotic cases were distinguished, as the latter clearly showed functional pattern induction.

In summary, black (including gray) and/or orange clusters of scales (*n* = 4), impairments of cover scales (*n* = 2), and orientation changes of scales (*n* = 1) were induced by Dll-GFP. The variability of responses obtained in these five cases of color pattern changes and an additional eight cases of necrotic black patches may be unavoidable in this experimental system, due to slight differences in the timing and location of infection, the amounts of infected virus, and the genetic backgrounds of host butterfly individuals. However, occurrence of scales showing element-like colors in background areas was a common feature associated with the *Dll-gfp* baculovirus infection.

## Discussion

The present study demonstrated that Dll can induce element-like colors (i.e., black, gray, and orange colors). It is important to note that the color patterns obtained here were so unique that they cannot be obtained from phenotypic plasticity or unrecognized physical or physiological “treatment”. It is also important to emphasize that high GFP expression alone did not result in any abnormal color patterns, consistent with the results of a previous study [31]. One possible phenotypic effect of the *gfp* baculovirus on wings was an exposure of the wing basal membrane without scales. Together with the third and fifth cases of the ectopic color patterns induced by the *Dll-gfp* baculovirus, the impairment of scale development may be a non-specific effect of baculovirus infection in wing areas in which the infection level is high.

There is no reason to believe that toxicity of Dll expression induces color pattern changes except necrotic damage. Color pattern changes and necrotic damage can be distinguished easily. Although there were only five induced cases without necrosis, we note that these cases share a common feature: occurrence of normal scales showing element-associated colors (black, gray, or orange) in background areas, suggesting that they are likely induced by exogenous Dll. In addition to the black and orange colors, Dll may also determine scale orientation, although we found only one case with rotated scales.

Heterogeneous infection in various parts of the pupae was observed, even at identical virus titers. The variability is unavoidable as it has been observed with baculovirus [31, 32], Sindbis virus [34], and other viral systems [35–37]. Thus, we believe that the common tendency is more important than the variation in the five induced cases.

Also noteworthy is the GFP fluorescent signals from scales themselves. This is surprising in that scales are extracellular structures and contain dark pigments that could prevent GFP from being fluorescent. This fact also indicates that our gene transfer method is as efficient as an alternative method that was reported recently [38].

Especially interesting are two cases in which black-orange clusters were induced. The adjacent placement of these two colors is an essential feature of eyespots in this species. No orange color is present on the normal dorsal hindwings, except in the eyespot ring. Failure to obtain a concentric eyespot-like structure may be due in part to variable infection of baculovirus in differentiating wing cells. Furthermore, other genes may be required to form a circular color pattern. In these two important cases, we did not observe direct GFP fluorescent signals. However, this is unsurprising given that scales are darkly pigmented extracellular structures.

The candidate genes for eyespot formation are expressed at prospective focus from the late larval to early pupal stages [9–16]. We were able to ectopically express Dll only after pupation; an injection at the prepupal stage almost always resulted in death of the treated individuals. We have not succeeded in the larval treatment, either. Although ectopic eyespots can be produced by physical damage on the pupal wing tissues in *J. orithya* and its related species a few days post-pupation [23–26], one technical concern is that GFP fluorescent signals were detected at the fourth day post-pupation; we thus confirmed RNA transcript for *Dll-gfp* in 4-day-old pupae. Hence, the time window of our experimental system did not completely overlap with the time window of endogenous Dll expression. This methodological constraint may be a reason why the induction efficiency for color pattern elements was low in the present study. Nonetheless, we were able to obtain color pattern changes. This fact may indicate that the pupal wing tissue at the fourth day post-pupation retains the ability to form eyespots. Alternatively, and in our view more likely, exogenous Dll may have been expressed immediately after infection, although not investigated in the present study, in which case exogenous Dll might have functioned within the time window for endogenous Dll expression.

In a previous transgenic study, Dll was reported to induce black scales [21]. In the present study, we also observed black cover scales functionally induced by Dll; these black scales were associated with orange or gray scales in three cases, and in an additional case, a large distinct cluster of gray scales was observed. In all of these four cases, the black or gray scales are unlikely to be caused by toxic effects of Dll. Importantly, Dll toxicity was clearly identified in the necrotic cases that we observed. The necrotic black patches were different from the elemental color pattern changes.

If the black scale induction is not a loss of cover scales in the previous transgenic study [21], the black scales [21] might have been caused simply by low Dll levels; Dll is able to induce other elemental coloration (i.e., a yellow ring in *B. anynana*), but the up-regulated Dll levels were insufficient to do so. This interpretation is consistent with the results of the present study, where high Dll levels induced both black and orange scales together in *J. orithya*. We believe for these reasons that the transgenic and baculovirus-mediated approaches reported here are complementary.

The results of the present study support the long-standing hypothesis that Dll plays an important role in eyespot formation. However, our experiments cannot directly test whether Dll is responsible for eyespot size. Dll may contribute to the elemental formation that is triggered by upstream signals in collaboration with other molecular factors because Dll is unlikely to be sufficient for eyespot formation. We believe that Dll is not sufficient for eyespot size determination, either [22]. Nonetheless, Dll induced element-like scales, demonstrating that it plays an instructive role in elemental formation.

The color pattern determination process is probably executed through a serial induction process that involves not only *Dll* but also many other genes. We believe that serial induction involving lateral inhibition may be a key mechanism for developing color patterns in butterflies [8, 39–44], as well as in fish [45]. Calcium signals that were recently discovered in pupal wings (and hence calcium-related genes) may also contribute directly or indirectly to color pattern determination [46].

## Conclusions

The ability of Dll to induce element-like color patterns in butterfly wings is demonstrated here for the first time. The present study suggests that Dll likely functions in specifying element-like color patterns in *Junonia* butterfly wings during development. However, Dll is unlikely sufficient to induce an entire eyespot, although it can induce black, gray, and orange colors in the wing.

### Availability of supporting data

Sequence data for the *Dll-gfp* construct are available from GenBank (Accession No. KP748528).

## Abbreviations

AcNPV: *Autographa californica* nucleopolyhedrovirus
Dll: Distal-less
GFP: Green fluorescent protein
*J.*: *Junonia*
